# Evaluation of Trap Systems for Monitoring of *Odontothrips loti* and *Frankliniella occidentalis:* A Pilot Field Trial

**DOI:** 10.3390/insects17010084

**Published:** 2026-01-11

**Authors:** Yingning Luo, Chen Han, Xiongbing Tu, Mark R. McNeill, Xuewei Yin, Liping Ban

**Affiliations:** 1 College of Grassland Science and Technology, China Agricultural University, Beijing 100193, China; ynieng_luo@163.com; 2 State Key Laboratory for Biology of Plant Diseases and Insect Pests, Chinese Academy of Agricultural Sciences, Beijing 100193, China; 3 Powerchina Huadong Engineering Corporation Limited, Hangzhou 311100, China; 4 Institute of Plant Protection, Chinese Academy of Agricultural Sciences, Beijing 100193, China; 5 Bioeconomy Science Institute, Christchurch 8140, New Zealand

**Keywords:** thrips, *Medicago sativa*, semiochemicals, integrated pest management, trap system

## Abstract

Plant-derived volatile organic compounds (VOCs) are widely used as insect attractants for population monitoring, offering an efficient and eco-friendly approach to pest management. Since thrips are the dominant pest species in alfalfa fields, this study aimed to identify a suitable attractant trap design that could be employed to monitor *Odontothrips loti* and *Frankliniella occidentalis*. The field experiment showed that *p*-Menth-8-en-2-one, dispensed through PE (Polyethylene) vials positioned at the top of the alfalfa canopy, attracted the most thrips, with the optimal concentrations of 1 µg/µL for *O. loti* and 50 µg/µL for *F. occidentalis,* respectively. When both species occur in alfalfa, PVC pipes dispensing *p*-Menth-8-en-2-one at a concentration of 1 µg/µL provide an effective attractant for both species, offering an indication of presence and relative abundance. Understanding the incidence and abundance of both species in the field provides growers an opportunity to target treatments to protect crops before significant damage occurs, reduce insecticide overuse, and support integrated pest management strategies for these two high-impact pests.

## Simple Summary

Thrips cause severe damage to alfalfa, leading to substantial production and economic losses. With chemical insecticides resistance escalating due to overuse, sustainable control alternatives are needed. We evaluated a novel trapping system using alfalfa-derived semiochemicals for thrips management. Our results demonstrated that white sticky traps baited with *p*-Menth-8-en-2-one dispensed through polyethylene vials, positioned at canopy height, captured the highest number of *Odontothrips loti* and *Frankliniella occidentalis*. This preliminary field trial offers a science-based approach to support timely decisions for implementing control strategies in the early stage before crop damage becomes significant and to reduce the number of insecticide applications required for control.

## 1. Introduction

*Medicago sativa* L. (alfalfa) is susceptible to infestation by thrips throughout its development. Key species include the genera of *Apterothrips*, *Frankliniella*, and *Odontothrips* [1,2,3]. *Odontothrips loti* (Haliday, 1852) (Thysanoptera: Thripidae) is an oligophagous insect specialized on legumes, with alfalfa as a primary host, and has an infestation rate exceeding 70% in the northwest of China [4,5]. The western flower thrips *Frankliniella occidentalis* (Pergande, 1895) (Thysanoptera: Thripidae) present a contrasting threat as a highly polyphagous and invasive pest, now globally distributed partly due to human activity [6,7]. Both species co-occur frequently in alfalfa. Substantial reductions in crop yield and quality accompany high population densities of thrips. Documented effects include elevated concentrations of tannin and lignin, coupled with suppressed plant height and diminished leaf area [8,9]. Annual yield losses directly attributable to thrips often exceed 20%, a consequence not only of direct feeding but also of their role as vectors for plant viruses such as alfalfa mosaic virus [10,11,12,13,14].

Effective thrips management is complicated by several intrinsic biological factors. These include rapid life cycles, high reproductive potential, cryptic habits, and a haplodiploid sex-determination system. These traits collectively promote the swift evolution of insecticide resistance, a problem exacerbated by heavy reliance on chemical controls [15,16]. Integrated pest management (IPM) strategies, which incorporate systematic pest monitoring, offer improved cost-efficiency over sole dependence on insecticides. Sticky traps baited with p-anisaldehyde, eugenol, farnesene, 3-methyl butanal, dodecyl acetate, and tetradecyl acetate have been reported to significantly raise the number of stick tea thrip *Dendrothrips minowai* caught in tea plantations, respectively [17,18]. The development of precise sampling methodologies remains a critical need, as they enable more targeted pesticide applications and can lead to significant reductions in control expenditures [19,20].

In a laboratory study, we identified the headspace volatiles of alfalfa plants and conducted a series of behavioral experiments, which found that the semiochemical *p*-Menth-8-en-2 was highly attractive to both *O. loti* and *F. occidentalis* [21]. The response of both *O. loti* and *F. occidentalis* to *p*-Menth-8-en-2-one ranged from 2.05 to 3.07 times greater than the control, respectively, with dispenser type and concentration acting as important variables in attraction [21]. Based on these results, a system for monitoring *O. loti* and *F. occidentalis* in the field was investigated in relation to dispenser type, placement height, sampling time, and *p*-Menth-8-en-2 concentration in this study. The aim is to develop a trap system that provides growers with accurate information of thrips occurrence and abundance, allowing them to schedule pest management strategies before significant yield losses occur.

## 2. Materials and Methods

### 2.1. Experimental Site

The study was carried out at the Shangzhuang Experimental Station of China Agricultural University (40°1′42″ N, 116°16′43″ E) in Haidian District, Beijing, from August 3 to 20 (summer) 2023. The alfalfa cultivar Zhongmu No.1 was sown in July 2023 and subsequently managed using standard farming practices from its establishment, with no pesticides applied prior to and during the trial. The field site covered an area of 1170 m^2^ (90 m × 13 m).

### 2.2. Field Trials

The presence of thrips prior to establishing the experiment was ascertained by gentle shaking of a subsample of alfalfa plants onto a funnel device made from A4 paper and a 50 mL centrifuge tube [22]. These were collected and identified to species under a binocular scope (10× magnification). This showed that the *Thrips* species occurring in the stand were dominated (90%) by both *Odontothrips loti* and *Frankliniella occidentalis*. These two species are easily distinguished by their morphology and color.

The field trials comprised two components which were run concurrently. For Experiment One, the effect of trap height on catch was determined using unbaited sticky traps. Traps were suspended from 1.8 m wooden poles at −20, −10, 0, 10, and 20 cm, measured from the bottom edge of the sticky board relative to the top of alfalfa canopy (0 cm). The sticky traps were double-sided, white, and 25 cm × 20 cm (Pherobio, Beijing, China), affixed to the wooden poles with wire, and distributed approximately 4 m apart.

The second experiment examined the effect of different dispensers and concentrations of *p*-Menth-8-en-2-one. Three dispensers were evaluated: PE vial (1 mL volume, Pherobio, Beijing, China), rubber plug (green hollow rubber, 20 mm × 9 mm × 6 mm, Pherobio, Beijing, China), and PVC pipe (white, 180 mm length × 5 mm diameter, Pherobio, Beijing, China). Each of the three dispensers were immersed in one of four concentrations of *p*-Menth-8-en-2-one (1, 10, 50 and 100 μg/μL) for 24 h, with paraffin oil as the control [23], air-dried in the laboratory for 2 h at 26 °C, sealed in a zip lock plastic bag (0.08 mm thick), and stored in a refrigerator at 4 °C until required (within 24 h). For placement in the field, the dispensers were stuck to the middle of the top third of each trap. The sticky traps were placed just above the alfalfa canopy and distributed approximately 4 m apart.

Boards were checked approximately every three days for 17 days from 3 to 20 August for a total of five times (except for day 9, due to prolonged rainfall). At each inspection, the sticky boards were replaced, and the same dispenser reattached (not replaced) to the new board. The boards collected at each inspection were returned to the laboratory and the number of thrips counted and their species identified by observing their morphological characteristics under a stereomicroscope (10× magnification). In summary, there were five trap heights in Experiment One, and three dispenser types × five concentrations in Experiment Two. All treatments were replicated three times, giving a total of 60 traps overall.

### 2.3. Data Analysis

We used IBM SPSS Statistics V22.0 (IBM, Armonk, NY, USA) to perform Multi-way ANOVA to detect differences in the amount of insects trapped, after clarifying the significance of the main effect and the interaction effect, multiple comparisons were performed by Duncan’s methods. Visualization was constructed by GraphPad Prism 10.1.1 (GraphPad Software, Boston, MA, USA).

## 3. Results

Overall, as the duration of the trapping setup time increased, the number of thrips also rose, which was largely consistent with population increase in relation to plant growth. In early August, persistent rain led to an apparent decrease in the number of thrips captured at day 6, and the timing of the third survey was postponed for an extra two days, with sampling carried out on day 11 (Table 1 and Table 2).

We assessed both the effect of trap height and effectiveness of *p*-Menth-8-en-2-one as an attractant in relation to dispenser type, and the *p*-Menth-8-en-2-one concentration. Multi-way ANOVA showed that there were significant effects of trap height and sample time for both species (all *p* < 0.01) (Table 1). There were also significant *Thrips* species × trap height (*p* < 0.01), *Thrips* species × sample time (*p* < 0.01), and trap height × sample time (*p* < 0.01) interactions. The interaction between *Thrips* species × trap height × time was not significant (Table 1).

For Experiment Two, there were significant (*p* < 0.05) responses by *Thrips* species, dispenser type, concentration, and sampling time. There were also significant *Thrips* species × dispenser type (*p* < 0.01), *Thrips* species × concentration (*p* < 0.01), *Thrips* species × sample time (*p* < 0.001), dispenser × concentration (*p* < 0.01), concentration × sample time (*p* < 0.01), *Thrips* species × dispenser × concentration (*p* < 0.01), *Thrips* species × concentration × sample time (*p* < 0.01), dispenser × concentration × sample time (*p* < 0.01), and *Thrips* species × dispenser × concentration × sample time (*p* < 0.01) interactions. The interactions between dispenser × sample time and *Thrips* species × dispenser × sample time was not significant (Table 2).

**Figure 1:**
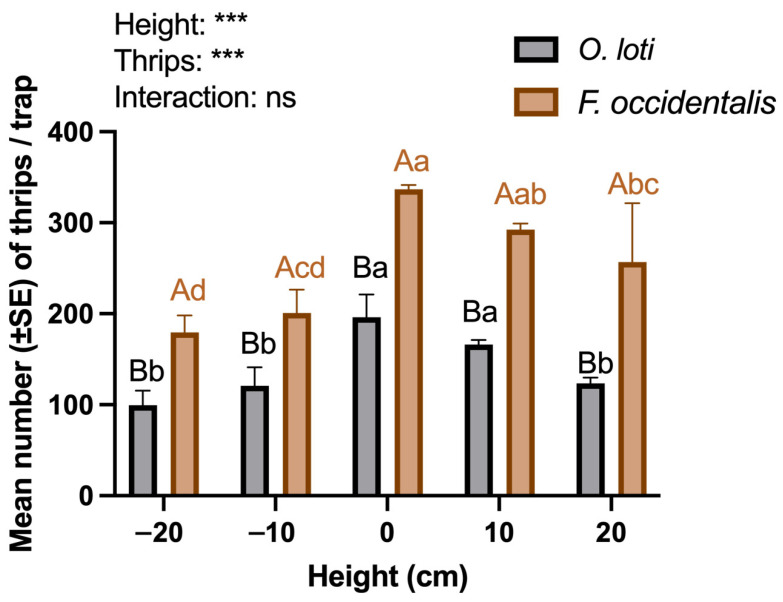
Mean (±SE) total number of thrips captured in relation to height of sticky boards. Height was measured relative to the bottom edge of the sticky board in relation to the top of alfalfa canopy (0 cm). Data were tested using a two-way ANOVA for factors height and thrip species, with different letters indicating Duncan’s multiple comparison of the differences in the number of thrips trapped under different height (lowercase) or species (uppercase). The overall significances in response to the two thrips and five placement heights are as follows: ns—no significant difference and *** *p* < 0.001.

When considering placement heights and thrip species, significant differences in trapping efficiency are also observed (*p* < 0.001, Figure 1). The highest number of both species were captured on traps suspended just above the alfalfa canopy height, although this was not significantly different from traps placed 10 cm above the canopy. Moreover, it was found that the number of *F. occidentalis* was consistently higher than that of *O. loti* across the different heights (*p* < 0.001, Figure 1).

**Figure 2:**
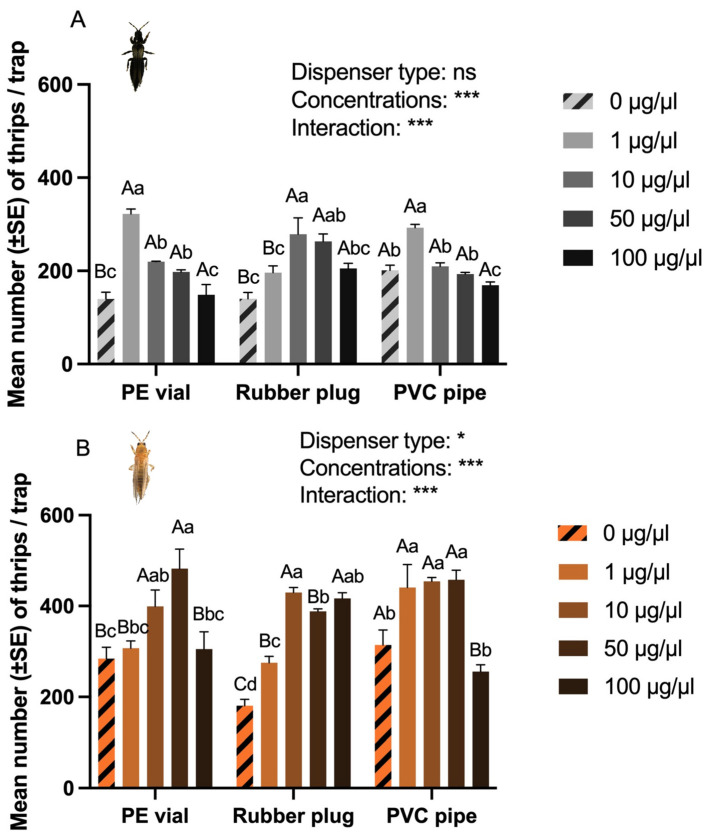
Effect of dispenser type and concentration (μg/μL) on the mean (±SE) capture of both *O. loti* and *F. occidentalis* in the field trial. (**A**) Number of *O. loti* captured, and (**B**) *F. occidentalis* captured, respectively, at the five concentrations and three dispenser types. Data were tested using a two-way ANOVA for factors dispenser type and concentration, with different letters indicating Duncan’s multiple comparison of the differences in number of thrips trapped under different concentrations (lowercase) or dispenser type (uppercase). The overall significances in response to the two *p*-Menth-8-en-2-one concentrations and three different dispensers are as follows: ns—no significant difference * *p* < 0.05 and *** *p* < 0.001.

When considering both dispenser type and concentration, it was found that dispenser type did not significantly affect the number of *O. loti* captured (*p* = 0.474, Figure 2A), but did significantly influence the number of *F. occidentalis* captured (*p* = 0.032, Figure 2B). Concentration significantly impacted the number of both *O. loti* (F = 28.780, *p* < 0.001) and *F. occidentalis* (F = 24.400, *p* < 0.001) captured, with higher numbers of *O. loti* caught in traps containing 1 μg/μL *p*-Menth-8-en-2-one. For *F. occidentalis*, the highest catches occurred at concentrations from 10 to 50 μg/μL (Figure 2B). There was also a significant dispenser type and concentration interaction for both *O. loti* (F = 10.920, *p* < 0.001, Figure 2A), and *F. occidentalis* (F = 6.992, *p* < 0.001, Figure 2B), respectively. PE vials dosed with a concentration of 1 µg/µL *p*-Menth-8-en-2-one were found to attract the highest number of *O. loti* (Figure 2A). Conversely, PE vials dosed with 50 µg/µL *p*-Menth-8-en-2-one attracted the highest number of *F. occidentalis* (Figure 2B).

## 4. Discussion

This study found that under field conditions, *p*-Menth-8-en-2-one was an effective attractant for both *O. loti* and *F. occidentalis*. Due to limitations related to the size of the field used in the study, the traps were placed 4 m apart. This would not be standard practice for field evaluations because of potential interference of between treatments in terms of odor flows across the site [24,25,26]. However, despite the close spatial layout of traps, significant differences were found in catches in relation to release rate and placement height, indicating that the results have validity.

Push–pull strategies involve the use of behavior-modifying stimuli to manipulate the distribution and abundance of both pest and beneficial insects, thereby reducing pest populations on the crop [27]. The innovative use of attractants or repellents has been demonstrated as a promising pest management tool for future farming systems. However, challenges such as high volatility and instability of these compounds need to be addressed for their effective application [28]. In this study, we trialed three dispenser types, and found that the PE vial was the most efficient release material. Similar results also observed with PE vial for *Apolygus lucorum* (Meyer-Dur) [29] and *Protaetia brevitarsis* Lewis [30]. Trap height also influenced field trapping effectiveness, with the optimal placement found to be at the bottom of the board aligned with the canopy of the alfalfa crop, or within 10 cm above. This is likely because new growth is primarily concentrated at the upper part of the alfalfa plant. This positioning enhances the perception of visual and olfactory stimuli by the thrips. Similarly, it has been found that the maximum number of *Thrips alliorum* (Priesner) caught was when the bottom of the sticky board was positioned in line with or just above the tops of scallions (*Allium fistulosum* L.) [31]. The higher number of *F. occidentalis* caught with the same *p*-Menth-8-en-2-one concentration compared to *O. loti* is likely due to the relative abundance of the two populations, consistent with Experiment One and sampling prior to establishing the trial showing that *F. occidentalis* was the dominant species.

For field application, the density of traps for monitoring needs further evaluation, although it has also been shown that multiple dispensers placed in relatively close to each other (5 m apart) are still less likely to interfere with each other [32]. A working system of traps for monitoring thrips to alert the farmer to their presence in the crop would be helpful; therefore, monitoring and the initiation of management options would be a logical extension of this research. This would include the number of traps required for monitoring, positioned in relation to the crop (i.e., margins of field, in the crop, upwind or downwind) and trap height [24,25,26]. Furthermore, given that the current trial was limited in its temporal scope, future work may benefit from establishing a longer monitoring period based on the release rate and efficacy of the dispensers over time, which can enhance practical utilization for growers. The species being targeted will also determine the *p*-Menth-8-en-2-one concentration used. For *O. loti* a concentration of 1 µg/µL was the most effective for monitoring, while for *F. occidentalis*, the optimal concentration was 50 µg/µL, with either a PE vial or PVC pipe suitable dispensers. If monitoring both species in a crop, either concentration will mean that one species will be under-represented in captures. Selecting a concentration of 1 µg/µL with a PVC pipe dispenser may be a suitable compromise, as while not capturing as many of both species, it will provide an indication of presence and relative abundance.

## 5. Conclusions

The plant volatile *p*-Menth-8-en-2-one has been identified in alfalfa and is significantly attractive to both *O. loti* and *F. occidentalis*. This proof-of-concept study showed that white sticky traps baited with PE vials containing *p*-Menth-8-en-2-one, positioned at the top or just above the alfalfa canopy, generally attracted the largest number of both *Thrips* species. However, dispenser type and release rate could be adjusted depending on species present in the field. We propose that traps using *p*-Menth-8-en-2-one can be effective in monitoring thrips, providing growers with information that allows them to make timely decisions on implementing control strategies before significant damage occurs. Further research should focus on optimizing trap placement, determining the appropriate number of traps, and establishing action thresholds in relation to crop management. These steps are essential for developing an efficient and ‘fit for purpose’ field monitoring system.

## Figures and Tables

**Table 1:** Mean (±SE) catch of both *O. loti* and *F. occidentalis* in relation to trap height of sticky boards at 3 to 17 days following deployment of traps.

*Thrips* sp.	Trap Height (cm)	Time					Significant Differences
		3 d	6 d	11 d	14 d	17 d	
*Odontothrips* *loti*	−20	10 ± 1	8 ± 0	14 ± 1	19 ± 1	48 ± 8	*Thrips* spp. ** Height (cm) ** Time (d) ** Thrips × Height ** Thrips × Time ** Height × Time ** Thrips sp. × Height × Time NS
	−10	13 ± 2	8 ± 0	16 ± 3	27 ± 3	56 ± 9	
	0	28 ± 1	15 ± 1	39 ± 4	37 ± 5	77 ± 9	
	10	20 ± 1	14 ± 3	30 ± 5	34 ± 2	68 ± 1	
	20	17 ± 1	11 ± 1	17 ± 1	27 ± 1	51 ± 5	
*Frankliniella* *occidentalis*	−20	22 ± 3	16 ± 3	36 ± 4	35 ± 3	71 ± 4	
	−10	25 ± 3	20 ± 3	35 ± 7	48 ± 1	73 ± 3	
	0	54 ± 1	30 ± 4	76 ± 6	79 ± 3	98 ± 6	
	10	35 ± 1	25 ± 0	81 ± 8	58 ± 8	93 ± 3	
	20	29 ± 8	20 ± 4	68 ± 16	57 ± 3	82 ± 8	

Note: Height was measured relative to the bottom edge of the sticky board in relation to the top of alfalfa canopy (0 cm). The results are the number of thrips trapped, presented as mean ± standard error; data were tested using multiple way ANOVA for factor time, *Thrips* sp., and concentration. The overall significances in response to different factors are as follows: NS—no significant difference, ** *p* < 0.01.

**Table 2:** Mean (±SE) catch of both *O. loti* and *F. occidentalis* in relation to dispenser type and *p*-Menth-8-en-2-one concentration at 3 to 17 days following deployment of traps.

*Thrips* sp.	Dispenser Type	Concentration (μg/μL)	Time					Significant Differences
			3 d	6 d	11 d	14 d	17 d	
*Frankliniella* *occidentalis*	PE vial	0	15 ± 1	13 ± 1	67 ± 1	86 ± 4	103 ± 25	Thrips ** Dispenser ** Concentration ** Day ** Thrips × Dispenser ** Thrips × Concentration ** Thrips × Time ** Dispenser × Concentration ** Dispenser × Time NS Concentration × Time ** Thrips × Dispenser × Concentration ** Thrips × Dispenser × Time NS Thrips × Concentration × Time ** Dispenser × Concentration × Time ** Thrips × Dispenser × Concentration × Time **
		1	19 ± 2	12 ± 2	48 ± 6	65 ± 5	163 ± 19	
		10	20 ± 1	23 ± 3	71 ± 9	90 ± 13	195 ± 29	
		50	26 ± 1	23 ± 0	82 ± 18	105 ± 18	247 ± 9	
		100	15 ± 2	11 ± 3	44 ± 15	70 ± 16	165 ± 6	
	Rubber plug	0	15 ± 2	14 ± 5	30 ± 4	46 ± 5	76 ± 6	
		1	11 ± 1	15 ± 2	56 ± 5	69 ± 2	125 ± 7	
		10	18 ± 2	11 ± 0	95 ± 3	105 ± 3	200 ± 6	
		50	20 ± 1	13 ± 1	62 ± 8	83 ± 2	210 ± 16	
		100	14 ± 4	15 ± 6	84 ± 4	123 ± 21	180 ± 8	
	PVC pipe	0	22 ± 3	23 ± 4	71 ± 15	87 ± 23	112 ± 9	
		1	24 ± 2	23 ± 4	64 ± 26	125 ± 26	205 ± 26	
		10	25 ± 1	28 ± 1	99 ± 14	116 ± 11	186 ± 29	
		50	22 ± 3	30 ± 3	94 ± 8	109 ± 3	203 ± 11	
		100	19 ± 3	27 ± 3	44 ± 3	68 ± 2	97 ± 16	
*Odontothrips* *loti*	PE vial	0	13 ± 0	8 ± 1	23 ± 6	36 ± 3	59 ± 7	
		1	17 ± 1	21 ± 3	62 ± 8	73 ± 9	149 ± 7	
		10	14 ± 2	15 ± 2	35 ± 4	60 ± 4	95 ± 1	
		50	14 ± 3	10 ± 1	37 ± 2	50 ± 1	87 ± 3	
		100	8 ± 1	8 ± 1	29 ± 5	37 ± 6	66 ± 11	
	Rubber plug	0	6 ± 1	8 ± 1	27 ± 3	36 ± 4	63 ± 6	
		1	8 ± 1	12 ± 3	39 ± 3	49 ± 3	88 ± 5	
		10	12 ± 3	10 ± 1	59 ± 9	69 ± 10	128 ± 19	
		50	7 ± 1	17 ± 2	59 ± 3	62 ± 7	118 ± 11	
		100	8 ± 2	11 ± 1	42 ± 3	55 ± 3	89 ± 6	
	PVC pipe	0	14 ± 1	8 ± 1	38 ± 3	47 ± 6	95 ± 5	
		1	17 ± 1	16 ± 4	58 ± 5	67 ± 2	134 ± 5	
		10	12 ± 0	12 ± 3	37 ± 3	54 ± 2	94 ± 2	
		50	10 ± 1	12 ± 1	35 ± 1	49 ± 1	87 ± 1	
		100	9 ± 2	10 ± 1	34 ± 1	41 ± 2	75 ± 3	

Note: The results are the number of thrips trapped, presented as mean ± standard error; data were tested using multiple way ANOVA for factors time, *Thrips* sp., dispenser type, and concentration. The overall significances in response to different factors are as follows: NS—no significant difference, ** *p* < 0.01.

## Data Availability

The data presented in this study are available within the article.
